# New Texture Descriptor Based on Modified Fractional Entropy for Digital Image Splicing Forgery Detection

**DOI:** 10.3390/e21040371

**Published:** 2019-04-05

**Authors:** Hamid A. Jalab, Thamarai Subramaniam, Rabha W. Ibrahim, Hasan Kahtan, Nurul F. Mohd Noor

**Affiliations:** 1 Faculty of Computer Science & Information Technology, University of Malaya, Kuala Lumpur 50603, Malaysia; 2 Faculty of Computer Systems and Software Engineering, University Malaysia Pahang, Kuantan 26300, Pahang, Malaysia

**Keywords:** image forgery, image splicing, fractional entropy, fractional calculus, discrete wavelet transform

## Abstract

Forgery in digital images is immensely affected by the improvement of image manipulation tools. Image forgery can be classified as image splicing or copy-move on the basis of the image manipulation type. Image splicing involves creating a new tampered image by merging the components of one or more images. Moreover, image splicing disrupts the content and causes abnormality in the features of a tampered image. Most of the proposed algorithms are incapable of accurately classifying high-dimension feature vectors. Thus, the current study focuses on improving the accuracy of image splicing detection with low-dimension feature vectors. This study also proposes an approximated Machado fractional entropy (AMFE) of the discrete wavelet transform (DWT) to effectively capture splicing artifacts inside an image. AMFE is used as a new fractional texture descriptor, while DWT is applied to decompose the input image into a number of sub-images with different frequency bands. The standard image dataset CASIA v2 was used to evaluate the proposed approach. Superior detection accuracy and positive and false positive rates were achieved compared with other state-of-the-art approaches with a low-dimension of feature vectors.

## 1. Introduction

**Figure 1:**
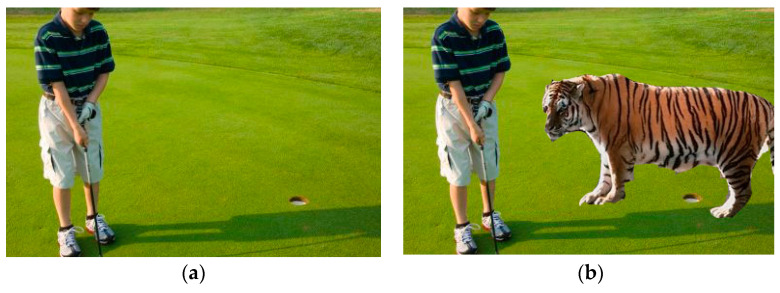
(**a**) Original image; (**b**) spliced image.

Image forgery detection refers to the process of identifying inconsistent regions in an image to authenticate the input digital image [1,2]. The detection of image forgery is divided into two types [3,4], namely active and passive authentication. The former depends on digital fingerprint and requires an original input image, whereas the latter is blind and does not require a priori knowledge about the original image. Image manipulation is classified into image splicing and copy-move. In image splicing, two images are combined to produce a new tampered image. Figure 1 displays an example of digital image splicing, in which (a) shows the original input image and (b) presents the tampered image with a region added from another image. The tiger that was added using an image editing tool introduced some inconsistencies in the tampered image contents, such as the sharp transience in the edges and texture patterns. Accordingly, the feature extraction approach should be robust to determine such changes in the image features. The most recent techniques of passive authentication for splicing forgery detection in digital images have common limitations that are related to the dimension of feature vectors and detection accuracy. The implemented techniques for detecting image splicing achieved good accuracy with different feature dimensions. However, achieving a high detection rate with a fairly small feature vector dimension cannot be guaranteed. To solve this problem, we propose a new fractional texture descriptor based on AMFE to capture the splicing artifact inside an image with low-feature dimension.

The remainder of this paper is organized as follows. Section 2 describes the material and methods. Section 3 explains the proposed method. Section 4 discusses the experimental results. Section 5 and Section 6 present a comparison with other methods and conclusions, respectively.

## 2. Materials and Methods

Numerous detection methods for image splicing have been implemented recently. The basis for such an implementation is the idea that image splicing often introduces some inconsistencies in the spliced image contents, such as the statistical characteristics of the images.

Zhao et al. (2011) proposed a new feature extraction approach based on the run length run number (RLRN) approach in four directions and support vector machine (SVM) as a classifier [5]. The proposed method applied four vectors with various directions of RLRN as feature vectors extracted for image splicing detection.

Moghaddasi et al. [6] enhanced the image splicing detection method on the basis of RLRN of the work of [5] using principal component analysis (PCA) and kernel PCA as dimension reduction methods. The experimental results showed that kernel PCA has the best result by using R, G, B, and gray-scale images to detect image splicing forgery.

Zhang et al. (2012) [7] developed a new splicing detection technique on the basis of a local binary pattern (LBP) operator, which is used to model 2D array components by applying the multiple size block approach of discrete cosine transform (DCT) (MBDCT) to the tampered images. To avoid the high dimensionality of the suggested features, PCA was used as a dimension reduction method.

Hakimi et al. (2015) [8] applied the YCBCR color channel to detect image splicing forgeries. The image features are extracted using the LBP operator of the wavelet transform of all none-overlapping image blocks, while the PCA was used as a dimension reduction method.

Park et al. (2016) [9] applied the characteristic function moments in the wavelet transform to extract image features and detect image splicing forgery. 

Recently, the fractional calculus and its applications was employed in different applications of signal and image possessing [10,11,12,13]. Rabha et al. (2015) [14] proposed new texture descriptors using fractional differential based on Machado fractional entropy. The experimental results indicated that the detection rates of image splicing were improved significantly compared with those of the existing splicing detection algorithms.

Shen et al. (2016) [15] proposed a passive image forgery detection method on the basis of the textural features based gray level co-occurrence matrices (TF-GLCM), on the difference block of the DCT arrays. The TF-GLCM texture descriptor was applied on the difference block DCT arrays. The statistical measures were applied to reduce the feature vector dimension and time complexity.

Li et al. (2017) [16] applied the Markov in quaternion discrete cosine transform (QDCT) to detect image splicing by capturing the inter-block correlation between the QDCT coefficients.

Salloum et al. (2017) [17] utilized a multi-task fully convolutional network (MFCN) to localize image splicing attacks. MFCN used two learning tasks to learn the label of the surface and the boundaries of the spliced regions. Experiments showed that the detection rate of this method is improved compared with the existing splicing detection and localization algorithms.

Moghaddasi et al. (2018) [1] used a low-dimensional singular value (SV) decomposition of the DCT coefficients to detect image splicing by computing the roughness measure of SVs. The dimension feature reduction was applied using the kernel PCA.

The preceding approaches focus on how to increase the accuracy of image splicing detection using different feature extractions and feature reduction methods. However, none of the aforementioned approaches have addressed the issue of considering high-detection accuracy without using the feature reduction approaches. Therefore, the current study focuses on improving the accuracy of image splicing detection with low-dimension feature vectors by developing a new fractional texture descriptor to effectively capture the splicing artifacts inside an image. This study also proposes an AMFE of the discrete wavelet transform (DWT) as a new fractional texture descriptor. Meanwhile, DWT is applied to decompose the input image into several sub-images with varying frequency bands.

## 3. Proposed Method

In this study, the proposed method comprises the following stages: pre-processing, feature extraction, and classification.

### 3.1. Pre-Processing

Two operations were applied in the pre-processing step; namely divide the image into none-overlapping image blocks and color space separation. The inconsistencies, which are caused by image splicing operations, are reflected in each image color space. Therefore, choosing the appropriate color space can play an important role in image splicing detection.

Numerous color spaces are offered for feature extraction. This study uses the YCbCr color spaces, which have been proven to be the best color space that reflects inconsistencies in the tampered image contents. The image block size applied in this study was experimentally fixed to 8 × 8 pixels.

### 3.2. Feature Extraction

Feature extraction has an important role in capturing the changes in the spatial distribution of images, a role that can be employed to achieve higher accuracy in detecting the spliced images. The texture features symbolize the information of the structure of images. In this study, we develop a new fractional texture descriptor based on the fractional entropy of the wavelet transform of all none-overlapping image blocks. The proposed feature extraction method is described as follows.

**Figure 2:**
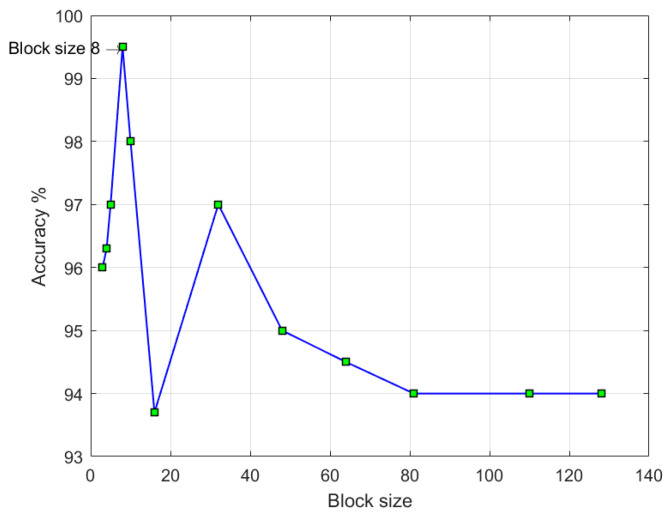
Block size with detection accuracy.

The first step is the transformation of the input image into the YCbCr color space. In the next step, the input image with each YCbCr color space is divided into non-overlap blocks with a size of 8 × 8 pixels. This size is proven experimentally as an optimal block size, as shown in Figure 2. The third step is the transformation of each image block into 2D DWTs. The fourth step is the AMFE feature extraction of the DWT coefficients using Equation (3). This step is an essential component of the study contribution. After these steps, the final features are fed into the SVM classifier to classify the input image into authentic or spliced image.

#### 3.2.1. Fractional Entropy

Through the times, researchers aim to generalize the concept of entropy, which is regularly used in several scientific disciplines. Entropy was presented in thermodynamics by Clausius and Boltzmann and later applied by Shannon and Jaynes in the information theory [18]. On the other hand, the application of fractional calculus has increased exponentially in almost all sciences. This progress in fractional calculus (differential and integral operators) leads to the extension of the concept of entropy into the fractional entropy, which showed an interested activity in complex dynamical systems [19,20,21]. Ubriaco [22] introduced the most important definition of fractional entropy, which is given by the following:

$$S_{\alpha }=\;\underset{k}{\sum }{\left[\;\left(-\ln\left(\rho_{k}\right)\right)\right]}^{\alpha }\rho_{k},\;\alpha \in \left[0,1\right].$$

Recently, Val´erio et al [23] made a generalization for the fractional entropy, using the fractional derivative operator of order α as follows:

(1) $$S_{\alpha }=\;\underset{k}{\sum }\left[\frac{-\rho_{k}^{-\alpha }}{\Gamma \left(1+\alpha \right)}\;\left(\ln\left(\rho_{k}\right)+\varphi \left(1\right)-\varphi \left(1-\alpha \right)\right)\right]\rho_{k},$$

where$\;\rho_{k}$ is the probability of occurrence of each pixel in an image block, φ(.) is the logarithmic derivative of the gamma function Γ, and *α* is the fractional parameter.

The local fractional calculus [24] provides a detailed information for images. Therefore, we employ this idea to generalize Equation (1) as follows:

(2) $$S_{\alpha }=\;\underset{k}{\sum }\left[\frac{-\rho_{k}^{-k\alpha }}{\Gamma \left(1+k\alpha \right)}\;\left(\ln\left(\rho_{k}\right)+\varphi \left(1\right)-\varphi \left(1-\alpha \right)\right)\right]\rho_{k}.$$

Equation (2) practically indicates a decrease from 1 to 1 − *α*, *α* ∈ (0,1), where *φ* decreases by 1/*α*. Therefore, we have derived the AMFE or modified fractional entropy as follows:

(3) $$S_{\alpha }\approx \underset{k}{\sum }\left[\frac{-\rho_{k}^{-k\alpha }}{\Gamma \left(1+k\alpha \right)}\;\left(\ln\left(\rho_{k}\right)+\frac{1}{\alpha }\right)\right]\rho_{k},\;\;\alpha \;\neq 0.$$

We were motivated by the method in the work of [14], which applied fractional calculus for image splicing detection and proposed a new fractional texture descriptor on the basis of AMFE for forgery detection in digital image splicing.

The suggested mathematical approach is used to calculate AMFE for each block on the basis of the frequency details of the input image to study the structure of the image.

This study extracts the AMFE value for each image block to reflect the changes in the suspicious image structure owing to image splicing.

The logic behind the preceding result is the ability of AMFE to preserve features in non-textured regions as well as to sharpen the texture detail. 

To extract AMFE from the input image, we first split the input image into block sizes of n × n pixels. The block size was properly selected to support the balance between detection accuracy and feature dimension. Although AMFE is unable to capture the artifacts caused by splicing in the case of small block sizes, the feature dimension increases in the case of the increasing block sizes. The appropriate block sizes empirically were selected as 8 × 8 pixels (see Figure 2).

#### 3.2.2. DWT

DWT is an extensively used tool in image processing. DWT decomposes an input image by applying low and high pass filters, thereby generating two coefficients. The approximation coefficient provides low-frequency image information, whereas the detail coefficient provides high-frequency image information.

This study uses one level of Daubechies wavelets “db1” D2 DWT. The majority of the image information is contained in the approximate coefficient of DWT. By contrast, the high-frequency coefficient values, which describe the edge and boundaries, are contained in the detail coefficients of the image.

### 3.3. Classification

The SVM is suggested in this study for image classification. The kernel used in SVM to compute the classifier is the fine Gaussian SVM [25]. The extracted features from the CASIA v2 dataset [26] are labelled as authentic and spliced images. The CASIA v2 image dataset is adopted to validate the proposed method. A 10-fold cross-validation method is used to estimate the accuracy of the proposed method. The image dataset is partitioned randomly into 10 folds that are approximately of equal size. Nine folds are used for training, while the remainder are used for testing. The proposed method was applied using MATLAB R2018b (MathWorks, Natick, MA, USA) on Windows 10 [25].

## 4. Experimental Results

The results are evaluated using the true negative (TNR), true positive (TPR), and average detection accuracy(ACC). TNR defines the percentage of negatives that are correctly classified as negative (for spliced images), whereas TPR defines the percentage of positives that are correctly classified as positives (for authentic images). 

**Figure 3:**
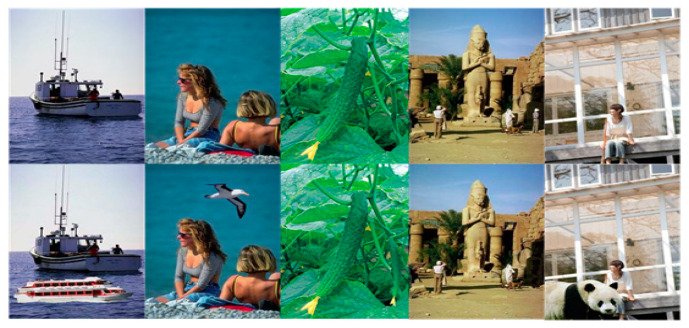
Samples of the images from CASIA v2.

The CASIA v2 image dataset [26] is used to evaluate the proposed method, which is widely used to detect image forgery and is publicly available. Figure 3 shows examples of the images from the image dataset.

**Figure 4:**
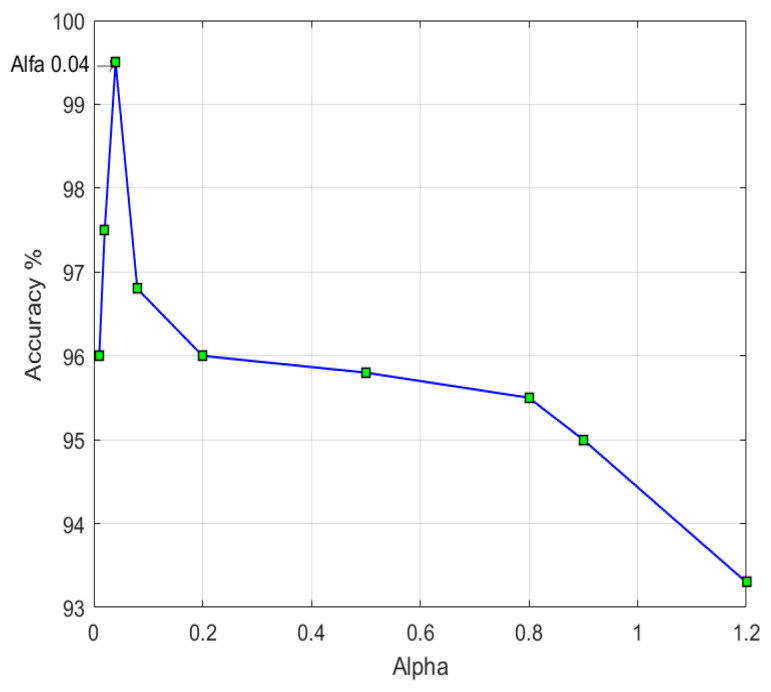
The value of α with detection accuracy.

In this study, the key parameter is *α*, in which the performance of the proposed AMFE texture descriptor changes on the basis of the *α* value. The values of *α* are linked to the AMFE process and are empirically selected to be equal to 0.04 (see Figure 4). The same value is considered for all experimentations in this study. Figure 4 shows the relation between detection accuracy and the value of *α* used in applying SVM as a classifier in CASIA v2 as the image dataset. The accuracy reached a peak value of 99.50% for *α* = 0.04.

The selection of a suitable color space is important in image splicing detection because the image splicing operation causes some variations in image features, which are reflected in each color space.

**Figure 5:**
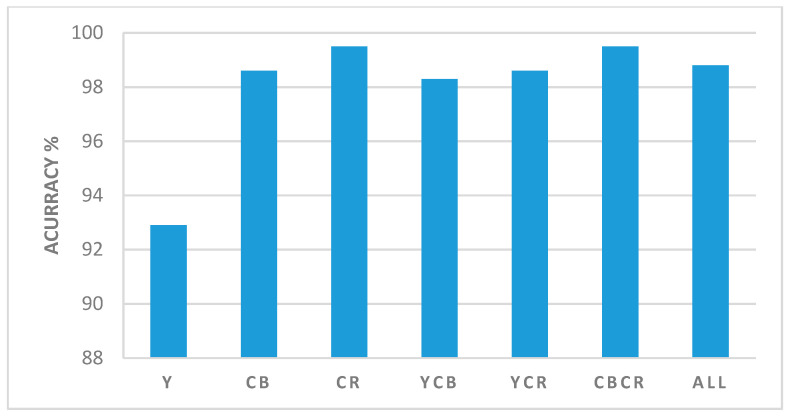
Color space combinations with detection accuracy.

This study selects YCbCr (Y (luma), Cb and Cr) as the color model. Figure 5 shows the color space combinations with detection accuracy. The highest detection accuracy of 99.50% is observed in Cr.

More experiments have been carried on to investigate the combination of two or three color spaces, such as CbCr, YCr, YCb, and YCbCr, on the proposed method. The highest detection accuracy of 99.50% was observed for the CbCr color channels.

Table 1 shows the results of the proposed approach without any feature dimension reduction. The main purpose of the dimension reduction is to reduce feature dimensionality by removing the redundant features in the feature vector. This study proposes AMFE to capture the splicing artifacts effectively without feature reduction, which is considered an essential component of this study’s contribution.

The highest detection accuracy is 99.50%, which was observed in Cr for 24 dimensions. The results of the detection accuracies from various color spaces verify that the proposed approach is more affected by Cr and Cb when compared with the other investigated color spaces. These results proved that the proposed AMFE is more sensitive to the chroma color spaces (Cb and Cr) than the luma (Y).

The performance is also evaluated by calculating TNR, which is the number of false negatives (splicing images), and TPR, which indicates the number of true positives (authentic images).

## 5. Comparison with Other Methods

The performance of the proposed approach is compared with other image splicing detection methods (see Table 2). The proposed approach achieved a relatively high average detection accuracy. The method of Zhao et al. [5] has the lowest accuracy with a dimensionality of 60. Although the algorithm of Moghaddasi et al. [1] has the highest accuracy with 60 dimension features, this approach applied the kernel PCA for feature reduction. The high accuracy using the Cr color space that reached 99.50% without feature reduction is the strength of the proposed method compared with the other methods using the CASIA v2 image dataset [26].

## 6. Conclusions and Future Research

Forged images that are created by image splicing are visually difficult to detect. Numerous splicing detection techniques may be affected by several problems, such as high feature dimensionality and low accuracy with high false positive rates. To solve these problems, image feature extraction should efficiently detect inconsistent regions in an image within low dimensionality. The new fractional texture descriptor based on AMFE was proposed to effectively capture the splicing forgeries inside the image. Therefore, the current study focused on evaluating the effectiveness of AMFE as a feature extraction approach for detecting image splicing. A set of experiments was designed to determine the effectiveness of the proposed AMFE method using the CASIA V2 image dataset. The results showed that the Cr color space has the best performance compared with those of the Cb and Y color spaces. The proposed approach achieved a relatively high average detection accuracy of 99.50% without any feature reduction, thereby proving the efficacy of applying AMFE in fractional calculus. Suggestions for future work include modifying the proposed approach to detect other types of image forgeries, such as copy-move forgeries.

## Figures and Tables

**Table 1:** Results of the proposed approach obtained from the CASIA v2 image dataset.

Colors	Dim	TNR%(Spl)	TPR%(Aut)	Accuracy %
Y	24	80	84	92.90
Cb	24	99	96	98.60
Cr	24	99	95	99.50
CbCr	48	99	95	99.50
YCb	48	99	97	98.30
YCr	48	99	99	98.60
All	72	99	98	98.80

**Table 2:** The results of the proposed approach compared with other methods.

Methods	Dimension Reduction	Dimension	TNR (%)	TPR (%)	Accuracy (%)
Zhao et al. [5] **Cr**	None	60	79.10	91.80	94.70
Hakimi. F et al. [8]	PCA	Not mentioned	Not mentioned	Not mentioned	97.21
Park et al. [9]	PCA	100	Not mentioned	Not mentioned	95.40
Shen et al. [15]	None	48	99.46	96.34	97.08
Moghaddasi et al. [1]	Kernel PCA	60	100	98.59	99.36
Proposed	None	24	99	95	99.50
